# 2332: Community engagement in clinical and translational research: A framework for research institutions

**DOI:** 10.1017/cts.2017.261

**Published:** 2018-05-10

**Authors:** Dennis P. Scanlon, Laura J. Wolf, Cynthia Chuang, Jen Kraschnewski, Eugene Lengerich, Susan McHale, Ian M. Paul, Janice Penrod

**Affiliations:** Penn State Clinical and Translational Science Institute, Hershey, PA, USA

## Abstract

OBJECTIVES/SPECIFIC AIMS: Community engagement is a commonly used term, but is complex in both meaning and application. In order to help academic institutions and administrators develop infrastructure to promote and support community engagement and to help investigators work productively with communities, this analysis discusses the major components of community engagement in research on both the institutional and individual project levels as well as the interplay between them. METHODS/STUDY POPULATION: A literature synthesis conducted by a community engagement in research committee at 1 CTSA institution that examined the myriad factors related to effective community engagement in research identified across multiple disciplines was used to distill the major factors identified, assesses the interplay of the identified factors, and produce a conceptual model to help administrators and investigators apply best practices in engaging communities in clinical and translational research. RESULTS/ANTICIPATED RESULTS: This work takes a concept—community engagement in research—that is often stated and discussed, but is highly complex and challenging to implement—and identifies and discusses the multiple, interrelated factors germane to it. The model illustrates that while community engagement in research is implemented in the context of individual projects, a deep and continual interplay between individual projects and the goals, capacity, and policies of research institutions is needed for rigorous, ethical, and effective community engagement. DISCUSSION/SIGNIFICANCE OF IMPACT: Results are presented through a conceptual framework which displays the major components needed for rigorous, ethical, and effective community engagement in clinical and translational research. In addition, the conceptual framework presented will provide assistance to those developing approaches to measure and evaluate institutional readiness for community engagement in research as well as the effectiveness of individual community engagement efforts.

